# Learning Outcomes of “GetSMART,” Education for Diagnostics and Targeted Treatment for HER2+ Metastatic Gastric and Colorectal Cancers

**DOI:** 10.1007/s13187-023-02384-8

**Published:** 2023-12-23

**Authors:** Monica Augustyniak, Emil Lou, Ginny Jacobs, Matthew Fleming, John Marshall, Anelisa Coutinho, Takayuki Yoshino

**Affiliations:** 1 AXDEV Group Inc, Brossard, QC J4W 3H2 Canada; 2 Masonic Cancer Center, University of Minnesota, Minneapolis, Minnesota USA; 3 AXDEV Global, Inc, Virginia Beach, Virginia USA; 4 Antidote Education Company, Dallas, Texas USA; 5 District of Columbia, Georgetown University, Washington, USA; 6 Multidisciplinary Oncology Institution, Clinica AMO, Salvador, Bahia, Brazil; 7 National Cancer Center Hospital East, Chiba, Japan

**Keywords:** Gastric cancer, Colorectal cancer, Human epidermal growth factor receptor 2 (HER2), Continuing medical education, Continuing health education, Program evaluation

## Abstract

The treatment landscape for patients affected by gastric and colorectal cancer (G&CRC) has significantly broadened over the past decade. Molecular diagnostic methods have improved with a precision oncology-driven approach to the development of treatment options tailored to specific molecular targets, including the human epidermal growth factor 2 (HER2). While scientific evidence on the role of HER2 in G&CRC has improved, there has been a lag in general understanding and applications of testing for HER2+ G&CRC and resulting targeting treatment in the wider oncology community. To better understand and address the root causes of this gap, a needs assessment deployed among 85 oncology care providers was conducted and informed the development of an accredited online educational program entitled “GetSMART.” The program consisted of four modules developed and narrated by experts in gastrointestinal oncology. The educational content and assessment metrics were guided by a confidence-based assessment (CBA) model and the Moore, Green, and Gallis outcomes framework. Assessment methods consisted of quantitative pre- and post-activity tests, an evaluation embedded within the education (*n* = 163), and semi-structured interviews (*n* = 5) post-activity completion. Findings indicated that “GetSMART” enhanced participants’ knowledge, confidence, and intent to change practice in relation to their (1) identification of HER2 aberrations, (2) selection of appropriate treatments for HER2+ G&CRC, and (3) ability to engage patients in shared decision-making and management of adverse events. “GetSMART” can therefore be a valuable educational resource for oncology HCPs caring for patients affected by HER2+ metastatic G&CRC, offering strategies to ensure an optimal team and patient-centered approach to the care being delivered.

**Supplementary Information:**

The online version contains supplementary material available at 10.1007/s13187-023-02384-8.

## Introduction

Gastric and colorectal cancers (G&CRC) are among the most incident and lethal cancers in the world [1]. When human epidermal growth factor 2 (HER2) is amplified or overexpressed, as is the case for about 20% of gastric cancers and 6% of colorectal cancers, there is an increased activation of cellular proliferation and differentiation pathways, tumor progression, metastasis, and resistance to treatment, which substantially affects the prognosis of patients with these cancers [2–4]. Increased HER2 signaling has been most widely associated with breast carcinomas, but a number of studies have shown similarly increased expression drives subsets of G&CRC.

Approaches to achieving accurate diagnosis and effective treatment for G&CRC have changed significantly over the past two decades, from the common use of immunohistochemistry (IHC) to the incorporation of fluorescence in situ hybridization (FISH), and also assessment of alterations using next-generation sequencing (NGS) [5]. For metastatic cases, improved molecular diagnostic methods accompanied by precision oncology-driven clinical trial designs have improved patients’ outcomes while more effectively tailoring treatment for individual patients [5]. The molecular testing of HER2, among other biomarkers, is strongly recommended in routine clinical practice to inform treatment decisions [6–9]. Despite increased evidence supporting routine evaluation and screening cases of HER2 amplification and respective use of targeted therapy in G&CRC, advances in this field have not been as readily recognized and adopted by healthcare professionals (HCPs) and the wider community [10].

An accredited online educational program entitled “GetSMART,” informed by a pre-intervention assessment survey, was deployed and evaluated to help address current educational gaps and needs of HCPs in the community. The program aimed to enhance learners’ knowledge and competencies in assessing and applying the molecular testing of G&CRC, including HER2 amplification and overexpression, prior to making an informed treatment decision with patients. The intended outcome was to help HCPs confidently offer an appropriate and balanced recommendation for the optimal treatment path of a patient with HER2+ mG&CRC. Critical components of the HCP’s communication with their patients would highlight the value of HER2 testing, the importance of accurate diagnosis and treatment selection, and communicate and address the need to skillfully monitor and manage adverse events (AEs) in the interest of optimizing safe and effective patient care. This paper presents the evaluation of the program, which aimed to assess the impact of “GetSMART” in terms of its educational outcomes and intent to change practice among learners when caring for patients with metastatic HER2+ G&CRC.

## Materials and Methods

### Program Description

The educational content of “GetSMART” was informed by (phase 1) a needs assessment survey administered to 85 HCPs caring for patients with HER2+ mG&CRC. Supplementary information A summarizes the methods and results of this survey in alignment with the final learning objectives of the program. The program was comprised of four interactive online modules relevant to the following areas of care: identification of HER2 aberrations, selection of appropriate treatment for HER2+ G&CRC, and patient engagement in shared decision-making (SDM) and management of AEs. Each module engaged learners in approximately 20–30 min of didactic content, in addition to case scenarios and questions aimed to elicit both discussion and reflection. The content was developed and presented by clinical experts (co-authors EL, JM, AC, and TY) to help learners apply their knowledge in practice and consider changes in their approach to patient care. Relevant educational resources on the various topics were presented as reinforcement materials and made available to learners for download. Learners could obtain CME credit after correctly responding to 75% of the case-based questions. The program was launched in January 2021 and made available to learners through April 2022.

### Evaluation Approach

A mixed-methods study design was used to evaluate (phase 2) the impact of the educational program. Methods included a (1) pre-post analysis of learners’ responses to matched assessment questions included in the educational activity; (2) descriptive analysis of learners’ responses to a formal evaluation immediately post-activity; and (3) qualitative interviews (20–30 min) with learners, at least 10 days after they had completed the activity. The evaluation was guided by the Moore, Green, and Gallis outcomes framework for planning and assessing continuing medical education (CME). The activity covered levels 1 (participation) to 5 (performance-self-reported) [11] and also included a confidence-based assessment (CBA) model to measure knowledge mastery [12]. To better understand the immediate impact of “GetSMART,” a comparison between pre- and (first attempt) post-activity assessment was decided.

### Participants

The potential learners of “GetSMART” were targeted via an international outreach campaign, targeting members of oncology teams (e.g., physicians, registered nurses, and advanced practice providers). For the evaluation, participants consisted of those learners who took part in all four modules of the activity, provided consent for including their data in the analysis, and/or agreed to participate in the interview.

### Data Collection

Data collection tools were developed in line with the guiding evaluation frameworks [11, 12], the learning objectives of the program, and the four targeted areas of care. Upon registration, learners were invited to provide their country, profession, specialty, and years of practice. Before and after completing modules, learners responded to knowledge-based multiple-choice questions and rated their confidence in response (1 = I’m guessing, 2 = I think, 3 = I’m sure). They were also asked to rate their perceived confidence (1 = not at all confident, 3 = neutral, 5 = very confident) and frequency (1 = never, 3 = sometimes, 5 = always) in performing specific behaviors at baseline and post-activity. A formal evaluation questionnaire was presented to learners immediately after completion of all four modules to collect learners’ perceptions of the program and intent to change specific elements in their individual and interprofessional practice. Learners were allowed to skip questions if they did not wish to respond. Interviews were conducted post-activity over a secure conference line (Zoom Video Communications Inc., 2022) by an expert moderator from AXDEV Group Inc. Questions were open-ended to elicit insights and reflections gained by learners, in addition to the anticipated impact on clinical practice, and remaining challenges and barriers to overcome. Interviews were recorded and transcribed.

### Analysis and Integration of Methods

The responses to open-ended questions and transcripts of interviews were coded and thematically analyzed in NVivo Version 12 (QSR International Pty Ltd., 2021) using an inductive reasoning approach that allowed the exploration of emerging trends and patterns [13] by a trained researcher at AXDEV Group Inc. Data derived from the quantitative assessment questions of learners were matched across data collection points (i.e., pre- and post-activity) and imported into SPSS Statistics (Version 27.0, IBM Corp., Armonk, NY, USA). Knowledge-based responses were dichotomized based on correctness and confidence in participants’ responses [12]. Participants who provided a correct response and were “sure” about it were classified as having achieved “mastery.” Confidence ratings of “5 = very confident” and “4 = somewhat confident” were grouped as “confident.” Frequency ratings of “5 = always” and “4 = often” were grouped as “always or often.” McNemar statistical tests were performed to measure the direction, magnitude, and significance of change between matched, dichotomized pre- and post-activity responses [14]. Descriptive analysis was performed on post-activity evaluation responses. Missing values were excluded. Quantitative and qualitative findings were linked to their respective learning objective and integrated based on their complementary nature to obtain a full understanding of the impact of “GetSMART” [15].

## Results

### Sample Size

Among the 284 learners who completed all four modules of the “GetSMART” program and its post-activity evaluation, 163 provided consent to have their data analyzed for reporting, of which 121 reported on their profession and specialty: 50% were registered nurses (RN), 18% physician assistants (PA), 14% medical doctors (MD), 6% pharmacists (PharmD), and 5% other. Specialties included oncology (32%), hematology/oncology (29%), general practice/internal medicine (11%), and other (28%). Of those who reported their country (*n* = 101), 92% were from the USA. The remaining participants opted out of the research study. Five qualitative interviews were completed with the following professions represented: RN specialized in oncology (*n* = 2), MD specialized in hematology/oncology (*n* = 1), MD specialized in gastroenterology (*n* = 1), and PharmD (*n* = 1), all from the USA.

### Main Findings

Pre-activity assessment questions of the learners who completed “GetSMART” confirmed that a majority had knowledge gaps when starting the activity (mean = 69% of learners not having selected accurate responses). Confidence pertaining to each of the learning objectives of “GetSMART” was low (mean = 71% of learners with a gap). The highest percentage of learners at baseline who demonstrated knowledge accuracy was 63%, specifically regarding the appropriateness of HER2 testing according to the NCCN guidelines for colorectal cancer. However, only 8% demonstrated knowledge mastery of this topic. An average of 59% of learners reported “never,” “seldom,” or “sometimes,” considering molecular testing results in the selection of treatment options for advanced G&CRC and 51% engaging patients with metastatic G&CRC in SDM.

Post-activity, “GetSMART” showed a significant impact (two-sided *p* < 0.05 for McNemar test) on the increased percentage of participants with a successful educational outcome (level 3 – knowledge, level 4 – confidence, and level 5 – performance) for many of the learning objectives (Tables 1, 2, and 3). The greatest gains were found for the percentage of learners post-activity with knowledge accuracy regarding the proportion of gastric cancers that are HER2-positive (+19%, *p* = 0.002, Table 1); knowledge mastery of AEs that should be monitored for patients receiving a HER2-targeted therapy (+21%, *p* < 0.001, Table 3); confidence identifying treatment for patients with advanced G&CRC based on molecular testing results (+35%, *p* < 0.001, Table 2); confidence anticipating and managing side effects related to HER2-targeted therapies (+16%, *p* < 0.001, Table 3); and intent to consider “always” or “often” HER2-targeted therapy as an option for patients with mG&CRC (+23%, *p* < 0.001, Table 2).

**Table 1.**
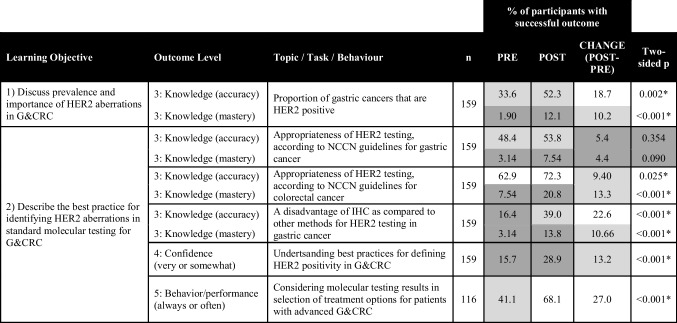
Learning﻿ objectives and educational outcomes pertaining to the identification of HER2 aberrations

This table presents the percentage of participants who demonstrated a successful educational outcome (knowledge accuracy, knowledge mastery, confidence, and intent to change) pre- to post-activity, with the two-sided *p*-value indicating significance if <0.05 for the McNemar statistical test. Percentage at pre and post in the range of 0–30% are shaded dark gray, 30–60% light gray, and 61–100% white. Changes pre to post in the range of 0–5% are shaded dark gray, 6–15% light gray, and >15% white. Successful outcomes were defined as having selected the accurate response (knowledge accuracy), having selected the accurate response and being sure about their response (knowledge mastery), responding very or somewhat confident on a self-rating scale (confidence), or selecting often or always on a self-reporting scale (behavior/performance)

**Table 2.**
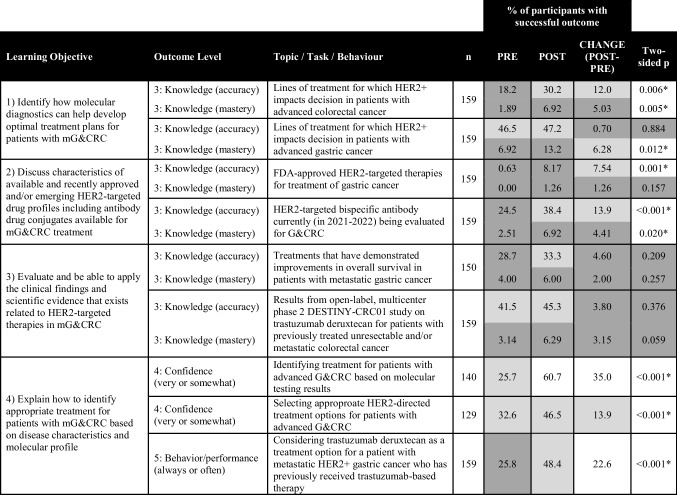
Learning﻿ objectives and educational outcomes pertaining to the selection of appropriate treatments for HER2+ G&CRC

This table﻿ presents the percentage of participants who demonstrated a successful educational outcome (knowledge accuracy, knowledge mastery, confidence, and intent to change) pre- to post-activity, with the two-sided *p*-value indicating significance if <0.05 for the McNemar statistical test. Percentage at pre and post in the range of 0–30% are shaded dark gray, 30–60% light gray, and 61–100% white. Changes pre to post in the range of 0–5% are shaded dark gray, 6–15% light gray, and >15% white. Successful outcomes were defined as having selected the accurate response (knowledge accuracy), having selected the accurate response and being sure about their response (knowledge mastery), responding very or somewhat confident on a self-rating scale (confidence), or selecting often or always on a self-reporting scale (behavior/performance)

**Table 3.**
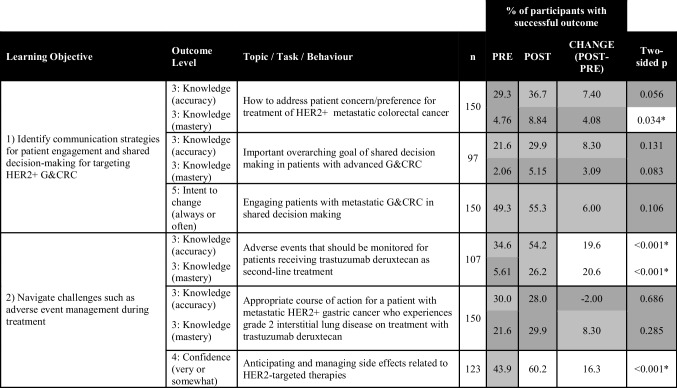
Learning objectives and educational outcomes pertaining to patient engagement in shared decision-making and management of adverse events

This table presents the percentage of participants who demonstrated a successful educational outcome (knowledge accuracy, knowledge mastery, confidence, and intent to change) pre- to post-activity, with the two-sided *p*-value indicating significance if <0.05 for the McNemar statistical test. Percentage at pre and post in the range of 0–30% are shaded dark gray, 30–60% light gray, and 61–100% white. Changes pre to post in the range of 0–5% are shaded dark gray, 6–15% light gray, and >15% white. Successful outcomes were defined as having selected the accurate response (knowledge accuracy), having selected the accurate response and being sure about their response (knowledge mastery), responding very or somewhat confident on a self-rating scale (confidence), or selecting often or always on a self-reporting scale (behavior/performance)

Evaluation-based responses showed that 82% of learners agreed that as a result of participating in “GetSMART,” they were better able to fulfill each of the learning objectives outlined in Tables 1, 2, and 3 (outcome level 4 – competency). In addition, 84% agreed that they were better able to recognize the barriers that prevent the acceptance or adoption of new clinical evidence into practice behaviors. A majority of learners (69%) projected a “moderate” or “major” impact on their performance (outcome level 5 – performance).

Reflections shared by participants who responded to open-ended questions of the evaluation and completed interviews emphasized the importance of leveraging a team-based approach to care when promptly ordering molecular test results, educating patients regarding the value of testing, and supporting an informed treatment decision. For example, an MD specialized in hematology/oncology mentioned: “*My Nurse Navigator, I basically empowered her* […] *that she automatically gets those tests sent off right away* […] *To make sure that nothing has slipped through the cracks. Before I had to order it specifically, and now, it’s getting automatically done.”* While an RN specialized in oncology reported in her evaluation to committing to the following as a result of attending the activity: “*Check to see if HER2 testing has been ordered on any new metastatic gastric patient that I meet for intake*.”

The remaining barriers or challenges that were noted by learners in their interviews were related to (a) patients resisting or refusing molecular testing due to low socioeconomic status and/or ability to afford medication; (b) complexities of obtaining financial reimbursement for certain molecular tests and targeted therapies by insurance companies; and (c) a need to ensure organizational processes are available to strengthen interprofessional collaboration when selecting treatment decisions and managing AEs. For example, an interviewed pharmacist mentioned: “*the nurses may not be as comfortable talking to patients about medications and explaining how to take it, or what reactions they might have* […] *We* [pharmacists] *can always be involved in the medication counseling part of it, regardless of what treatment it is.”* In comparison, an MD specializing in gastroenterology shared: “*two opinions are always better than one opinion. And if I miss something, somebody else will remind me about it. The program has stressed the importance of collaboration*.”

## Discussion

“GetSMART” informed HCPs, mostly located in the USA, involved in the care of patients affected by G&CRC. The program employed a survey to identify the gaps that were most crucial to address, informing the development and refinement of the program’s learning objectives and educational content. It engaged a range of professions, including mostly registered nurses, who play a central role in patient communication, coordination, and education, and can influence diagnostic and treatment decisions during tumor board discussions. The baseline assessment embedded within “GetSMART” confirmed the findings of the survey, appropriately addressing identified knowledge and confidence gaps related to the (1) identification of HER2 aberrations, (2) selection of appropriate treatments for HER2+ G&CRC, and (3) patient engagement in SDM and management of AEs.

In line with the Moore, Green, and Gallis (2009) framework [11], this mixed-methods evaluation assessed changes in educational outcomes at multiple levels: knowledge, competencies, and performance, which aim to improve patient outcomes (i.e., higher-level outcomes). The results obtained from the evaluation of “GetSMART” showed a remaining need to enhance clinicians’ knowledge in relation to precision-medicine and team-based care affecting patients with G&CRC due to HER2+ status, which suggests that current interventions focusing on knowledge acquisition are appropriate. Indeed, changes in skill, performance, and patient outcomes are possible after a thorough understanding of the foundational evidence in the field is established (i.e., clinicians’ knowledge is current and reflective of scientific advancements). After this initial step of performance improvement is achieved, there is an opportunity to incorporate additional strategies into performance improvement efforts, such as guided action plans with audit feedback.

The interprofessional nature of oncology care and the need to engage learners from multiple disciplines in interventions, such as “GetSMART,” is especially worth highlighting. While this concept equally applies to therapeutic areas outside of oncology, we should remind ourselves that patients affected by an illness as severe as cancer need to rapidly grasp the state of their health to be able to properly engage in a treatment and management plan most suitable to their needs, preferences, and socioeconomic circumstances (e.g., access to insurance coverage or ability to pay for the cost of molecular tests and targeted therapies). Consistent messaging from all professionals and specialists involved in the care of affected patients, who offer them evidence-based recommendations in line with their expected roles and responsibilities, is critical to ensure patients are knowledgeable of the best diagnostic and therapeutic options available to them. As demonstrated by the results of this study, patients facing high out-of-pocket costs may be reluctant to undergo molecular tests, even if recommended by their HCP. Hence, the involvement of social workers and nurses in diagnostic and treatment decisions affected by patients’ insurance coverage and/or other types of financial assistance programs (e.g., Medicare and Medicaid) is necessary. Future interventions should consider developing activities aimed at facilitating communication and collaboration among professionals and specialists involved in HER2+ G&CRC, especially at the metastatic stage. This could be achieved through a revision of organizational processes by clinical leaders and facilitated multidisciplinary team discussions related to points during which communication and collaboration between all professions and specialties can be enhanced. A limitation of this study was the smaller participation rate of gastroenterologists and oncologists, groups that are vital and most likely to influence diagnostic testing, therapeutic decision-making, and communications with this patient population. Additional efforts should be made to effectively inform this group of HCPs pertaining to available educational activities, such as “GetSMART” and future activities alike.

## Conclusion

“GetSMART” provided a foundation for addressing the pressing needs of HCPs involved in the care of patients affected by G&CRC due to HER2 aberrations. The intervention showed a positive impact on knowledge and confidence gains, in addition to anticipated performance in clinical practice that should enhance the health outcomes of patients affected by HER2+ G&CRC at the metastatic stage. The identification of HER2 aberrations in metastatic G&CRC and its appropriate treatment selection and management of AEs remains an ongoing challenge in oncology care that requires continued efforts to remain up-to-date regarding the value of testing. HCPs should be mindful of how to take steps to ensure an optimal team and patient-centered approach to care is being delivered to their patients.

### Supplementary Information

Below is the link to the electronic supplementary material.

Supplementary file1 (DOCX 101 KB)

## Data Availability

Anonymized datasets used/analysed for this article can be made available upon reasonable request to the corresponding author.
