# Supplementary material for: Learning Outcomes of “GetSMART,” Education for Diagnostics and Targeted Treatment for HER2+ Metastatic Gastric and Colorectal Cancers
Source: J Cancer Educ. 2023 Dec 23;39(2):118–25. doi: 10.1007/s13187-023-02384-8 (PMC10995009; doi:10.1007/s13187-023-02384-8)
Supplement: Supplementary file 1 — Supplementary file1 (DOCX 101 KB) [file 13187_2023_2384_MOESM1_ESM.docx]

**Supplemental material A – Needs Assessment Survey Informing “GetSMART”**

In June 2021, a pre-assessment survey was deployed internationally amongst medical oncologists, registered nurses (RNs), nurse practitioners (NPs) and physician assistants (PAs) caring for patients with metastatic gastric or colorectal cancer (mG&CRC) with HER2 aberrations. The survey, which consisted of 12 questions and a total of 33 items, was designed to examine the barriers and challenges that healthcare professionals (HCPs) face when caring for patients with mG&CRC related to molecular testing, treatment, management of adverse events and patient engagement. The purpose of this survey was to:

- To determine baseline knowledge of HER2+ drug profiles, their familiarity with current evidence, and confidence applying this information in clinic practice decisions, among an audience of potential learners
- To inform the educational content of “GetSMART”

**Inclusion criteria**: medical oncologist, RN, NP or PA specialized in oncology, with a minimum of 3 years of experience in current practice, spending at least 50% of professional time in patient care, and have a monthly case of patients with metastatic HER2+ G&CRC.

**Results**: A total of 85 respondents completed the survey. Mainly medical oncologists (95%) having 3-10 years of experience (n=50), from the United States (n=32) and Europe (n=27), see Figure 1. Table 1 shows main challenges and barriers identified, and how they informed the learning objectives of “GetSMART”.


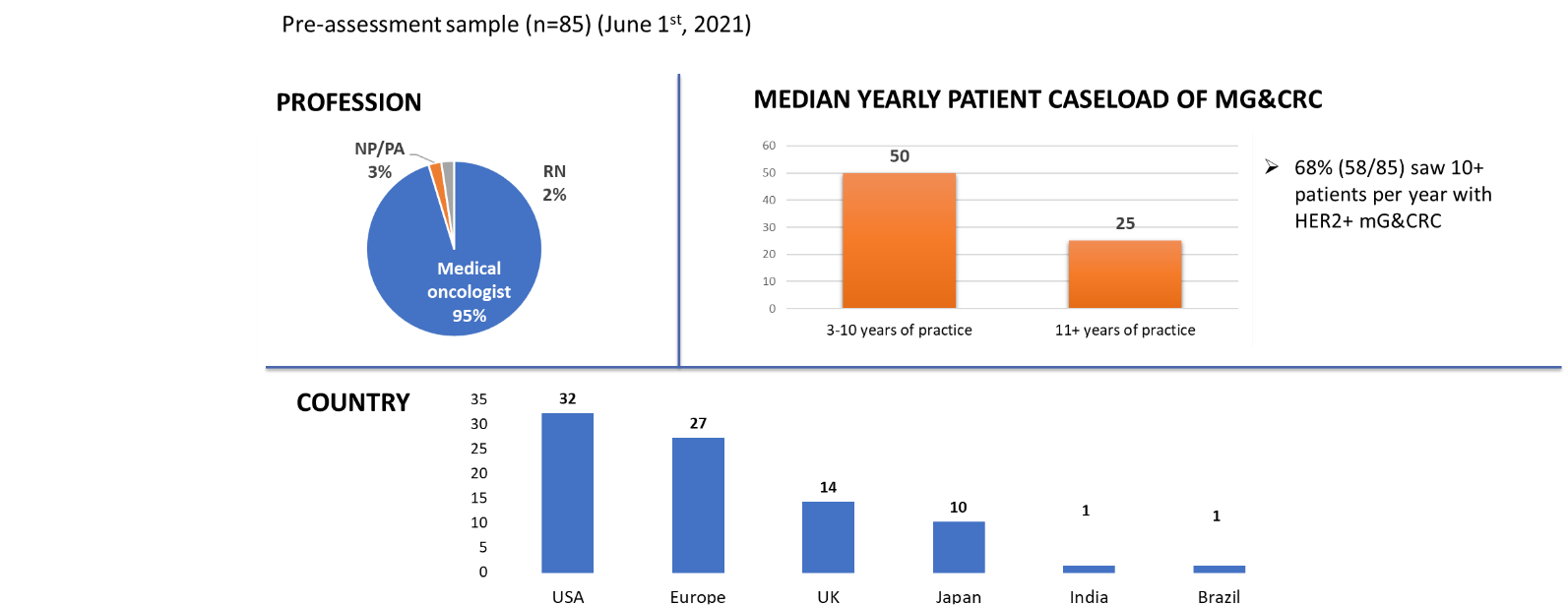


**Figure 1 - Demographics**

**Table 1 - Key findings from needs assessment survey informing learning objectives of "GetSMART"**

| **Area of challenge/barrier** | **Key findings** | **Learning objective of “GetSMART”** |
| --- | --- | --- |
| Identification of HER2 aberrations through appropriate molecular testing | *1/2 HCPs agreed with: “****challenges*** ***of using HER2 testing*** *in the care of patients with G&CRC* ***outweigh*** ***the benefits****”*  *Why?*   - - *Few patients effected by HER2+*   - *Out-of-site laboratories create delays*   - *Comments requesting “clear indication for tests”*   - *Suboptimal confidence using molecular testing amongst HCPs longer in clinical practice (i.e., out of school)* | Discuss the **prevalence and importance** of HER2 aberrations  Describe best practice for **identifying HER2 aberrations** |
| Treatment decision-making and application of available evidence related to HER2-targeted therapies | *1/3 HCPs reported a difficulty* ***selecting and sequencing treatment*** *for various patient profiles*  *Why?*   - - *Difficulty keeping up with all the available and emerging treatment options, including evidence*   - *Suboptimal confidence (<70%) selecting treatment for patients with multiple comorbidities*   - *Comments requesting “clear treatment algorithms”*   - *Reimbursement issues* | Identify **appropriate treatment choices**  Discuss characteristics of **HER2-targeted drug profiles**  Evaluate and willing to **apply scientific evidence** |
| Patient engagement in shared decision making  Management of adverse events in treatment of mG&CRC with HER2-targeted therapies | *1/3 HCPs reported a difficulty selecting and sequencing treatment* ***for various patient profiles***  *Why?*   - - *Suboptimal confidence (<70%) utilizing decision aids to explain treatment option to patients*   - *For HCPs with fewer than 25 patients per year, 30% noted a need for better awareness of strategies to engage different patients*   - *Patient education material (especially related to the availability of targeted therapies for various patient profiles) are needed* | Identify **effective communication strategies**  Navigate challenges such as **adverse event management** |

In conclusion, the needs assessment survey identified urgent educational needs as:

- 1. Identification of HER2 aberrations through appropriate molecular testing
  2. Treatment decision-making and application of available evidence related to HER2-targeted therapies
  3. Patient engagement in shared decision making
  4. Management of adverse events in treatment of mG&CRC with HER2-targeted therapies
